# Early multi-system physiological biomarkers of bronchopulmonary dysplasia in preterm infants: a matched case-control study based on bedside monitoring

**DOI:** 10.3389/fmed.2026.1902303

**Published:** 2026-07-27

**Authors:** Yu Ma, Jinhui Hu, Juan Liu, Yixin Zhang, Zhaojun Pan

**Affiliations:** Neonatal Medical Center, Affiliated Hospital of Yang Zhou University Medical College, Huai’an Maternal and Child Health Care Center, Huai’an, China

**Keywords:** bronchopulmonary dysplasia, echocardiography, oxygenation, perfusion index, preterm infant, risk prediction

## Abstract

**Objective:**

To identify early multi-system physiological biomarkers that can be obtained at the bedside prior to the formal diagnosis of bronchopulmonary dysplasia (BPD).

**Methods:**

We conducted a single-center retrospective 1:1 matched case-control study enrolling preterm infants of less than 32 weeks’ gestational age admitted to the neonatal intensive care unit (NICU) of our institution between June 2022 and June 2025. Infants with confirmed BPD were assigned to the BPD group, and non-BPD controls were matched 1:1 by gestational age and birth weight. Baseline characteristics, bedside monitoring parameters, blood gas indices, and quantitative echocardiographic features were compared between the two groups. Candidate early variables associated with BPD were first screened using LASSO-penalized logistic regression, after which representative indicators from each physiological domain were selected to construct a multivariate logistic regression model.

**Results:**

A total of 50 infants were included in each group, and no significant differences in baseline or maternal perinatal characteristics were found between the two groups (all *P* > 0.05). Multivariate analysis identified six independent predictors of BPD: invasive mechanical ventilation (IMV) [adjusted odds ratio (aOR): 21.312, 95% CI: 2.108–36.296, *P* = 0.023], peak FiO_2_ (aOR: 1.404, 95% CI: 1.022–1.931, *P* = 0.036), lower PI trend slope (aOR: 0.041, 95% CI: 0.002–0.974, *P* = 0.048), lower PaO_2_/FiO_2_ ratio (aOR: 0.026, 95% CI: 0.001–0.848, *P* = 0.040), lower LVEF (aOR: 0.008, 95% CI: 0.000–0.602, *P* = 0.028), and thinner IVSd (aOR: 0.043, 95% CI: 0.002–0.933, *P* = 0.045) as independent predictors of BPD. The combined model achieved excellent discrimination for BPD with an AUC of 0.982 (95% CI: 0.963–1.000), substantially exceeding the discriminative performance of each individual marker (individual AUC range: 0.625–0.864). At the optimal Youden index threshold, the model achieved a sensitivity of 93.47% and a specificity of 94.62%

**Conclusion:**

An early six-variable physiological profile integrating IMV, peak FiO_2_, PI trend slope, PaO_2_/FiO_2_ ratio, LVEF, and IVSd may facilitate early identification of preterm infants at increased risk of BPD.

## 1 Introduction

Since bronchopulmonary dysplasia (BPD) was first described more than half a century ago (1), it has remained the most common chronic complication of extremely preterm birth. Despite considerable advances in antenatal and neonatal intensive care that have improved overall survival, the disease burden of BPD has not declined proportionally (2, 3), and it continues to be closely associated with prolonged respiratory support and adverse long-term respiratory and neurodevelopmental outcomes (4). BPD is increasingly recognized as a multi-system disorder rather than a condition confined to the lung parenchyma (2). Injury to the developing pulmonary circulation can lead to pulmonary vascular disease and pulmonary hypertension, while also affecting the immature myocardium. These abnormalities may emerge early after birth and independently predict subsequent BPD and adverse outcomes (5–7). Taken together, these observations suggest that cardiovascular and hemodynamic derangements are present during an early intervention window when preventive strategies may still be effective.

Current BPD prediction tools, however, largely rely on gestational age, birth weight, and trajectories of respiratory support and oxygen requirement, with only moderate discriminative performance. Two major limitations characterize these tools: they incorporate little of the cardiovascular, microcirculatory, and metabolic physiological information obtainable at the bedside, and they rarely integrate information across organ systems (8, 9). In routine clinical practice, several routinely available bedside monitoring and laboratory modalities are readily accessible: the perfusion index (PI) and pleth variability index (PVI) derived from the pulse oximetry waveform, arterial blood gas-derived PaO_2_/FiO_2_ ratio and lactate, and functional echocardiography. These individually reflect peripheral perfusion, gas exchange efficiency, tissue metabolic status, and cardiac function (10), yet they are seldom measured concurrently or integrated into a unified early multi-system risk profile for BPD. The early multi-system physiological disturbances that accompany evolving BPD therefore remain underutilized in current risk stratification.

To address this gap, we conducted a gestational age- and birth weight-matched case-control study in preterm infants born before 32 weeks’ gestation. The primary aim was to identify early physiological biomarkers across the domains of peripheral perfusion, oxygenation, acid–base and metabolic balance, and cardiac function that could be obtained from routine bedside monitoring, blood gas analysis, and functional echocardiography and reliably distinguish infants who subsequently developed BPD from matched controls. A secondary aim was to select the most informative and mutually independent markers from these domains and combine them into a parsimonious joint model for early risk stratification.

## 2 Materials and methods

### 2.1 Study design, setting, and ethics

This was a single-center, retrospective, 1:1 matched case-control study conducted using the electronic medical record system of the NICU at Huai’an Maternal and Child Health Hospital. The study enrolled preterm infants of less than 32 weeks’ gestational age admitted to the NICU between June 2022 and June 2025. This study was approved by the Ethics Committee of Huai’an Maternal and Child Health Care Center (approval number: 2026007), and informed consent was obtained from the guardians of all participating neonates, and granting permission for the publication of their child’s clinical data. The study process adhered to the principles outlined in the Declaration of Helsinki and its subsequent amendments.

### 2.2 Case definition, matching, and eligibility criteria

Infants with a confirmed diagnosis of BPD according to the 2018 NICHD revised criteria (4) were enrolled in the BPD group. To minimize confounding by the two major determinants of BPD risk, gestational age and birth weight, controls were selected from the same admission period using 1:1 nearest-neighbor matching with calipers of ± 1 week for gestational age and ± 150 g for birth weight. Respiratory status and oxygenation parameters were not included as matching variables because they may represent early physiological features associated with subsequent BPD development. This process yielded 50 BPD cases and 50 matched controls (total *n* = 100).

Infants were excluded if they met any of the following criteria: (1) major congenital cardiac malformations or pulmonary developmental abnormalities; (2) confirmed genetic metabolic disorders, chromosomal abnormalities, or monogenic diseases; or (3) missing key clinical information that could affect outcome assessment or introduce potential bias.

### 2.3 Data collection, monitoring equipment, and quality control

Two trained investigators independently extracted objective indicators from electronic medical records across the following domains: (1) baseline, perinatal characteristics, major treatments, and neonatal complications, including gestational age, birth weight, sex, multiple gestation, antenatal corticosteroid exposure, surfactant administration, respiratory support modalities, including invasive mechanical ventilation (IMV) and continuous positive airway pressure, duration of invasive ventilation, peak FiO_2_, patent ductus arteriosus (PDA), atrial septal defect, ventricular septal defect, late-onset sepsis, and other clinically relevant comorbidities; (2) vital signs (baseline heart rate, SpO2, temperature, and respiratory rate); (3) pulse perfusion and microcirculatory indices, including daily PI values over the first 7 postnatal days and their 7-day mean, minimum, coefficient of variation, and trend slope, together with the 7-day mean, peak, and standard deviation of the PVI, SpO2 variability, and capillary refill time; (4) blood gas analyses, comprising measurements obtained immediately after birth and the most abnormal values recorded during the first 7 postnatal days, including minimum pH, most negative base excess (BE-B), minimum actual bicarbonate, maximum PCO2, minimum PO2, and peak lactate, together with the calculated PaO_2_/FiO_2_ ratio. Arterial blood gas analysis was performed routinely according to NICU clinical protocols in preterm infants requiring respiratory support. Because direct PaO_2_ measurements were available for all enrolled infants, the PaO_2_/FiO_2_ ratio was selected as the primary oxygenation index. The SpO2/FiO_2_ ratio was not routinely calculated or recorded in the electronic medical record during the study period and was therefore not included in the analysis; and (5) quantitative echocardiographic parameters, specifically LVEF, left ventricular fractional shortening (LVFS), left ventricular end-diastolic diameter (LVEDD), left ventricular end-systolic diameter (LVESD), interventricular septal thickness in diastole (IVSd), left ventricular posterior wall thickness in diastole (LVPWd), maximum ductus arteriosus flow velocity (Vmax), and pressure gradient.

Continuous pulse perfusion and variability indices were collected using a pulse oximeter (Philips SureSigns VM6). Echocardiographic measurements were performed by a credentialed sonographer using standard imaging planes. Outcome classification (BPD grading) was carried out by an independent assessor who was blinded to exposure data. The two data extractors cross-checked their entries and referred any discrepancies to a third-party adjudicator. All echocardiographic studies were conducted during a standardized window of postnatal days 3–7, during clinically stable periods (defined as absence of changes in ventilatory support settings within the preceding 12 h).

### 2.4 Statistical analysis

Statistical analyses were performed using R and Python. Continuous variables were assessed for normality using the Shapiro–Wilk test and are presented as mean ± standard deviation or median (P25, P75), as appropriate. Between-group comparisons were performed using the independent-samples *t*-test, Welch’s *t*-test, or Mann–Whitney U test. Categorical variables are presented as counts and percentages and were compared using the chi-squared test or Fisher’s exact test.

Variables available before formal BPD diagnosis and associated with BPD in univariate analyses were entered into LASSO-penalized logistic regression. The penalty parameter was selected by 10-fold cross-validation using the λ1se criterion. Variables with non-zero coefficients were grouped into physiological domains, and the variable with the largest absolute LASSO coefficient in each domain was retained to limit model complexity. Peak lactate was selected for the acid–base/metabolic domain because pH and BE-B were strongly correlated (*r* = 0.74). IMV and peak FiO_2_ were additionally included as prespecified indicators of respiratory disease severity. Pairwise correlations and variance inflation factors were examined to assess multicollinearity.

A Firth bias-corrected logistic regression model was fitted using the selected predictors. Continuous variables were standardized, and adjusted odds ratios are reported per one-standard-deviation increase. Model discrimination was evaluated using the area under the receiver operating characteristic curve, with bootstrap-derived 95% confidence intervals. Internal validation with 1,000 bootstrap resamples was used to estimate optimism-corrected AUC. Apparent calibration was assessed using the Hosmer–Lemeshow test, and Nagelkerke R^2^ was reported. The optimal classification threshold was determined using the maximum Youden index. All tests were two-sided, with *P* < 0.05 considered statistically significant.

## 3 Results

### 3.1 Baseline clinical characteristics

**FIGURE 1:**
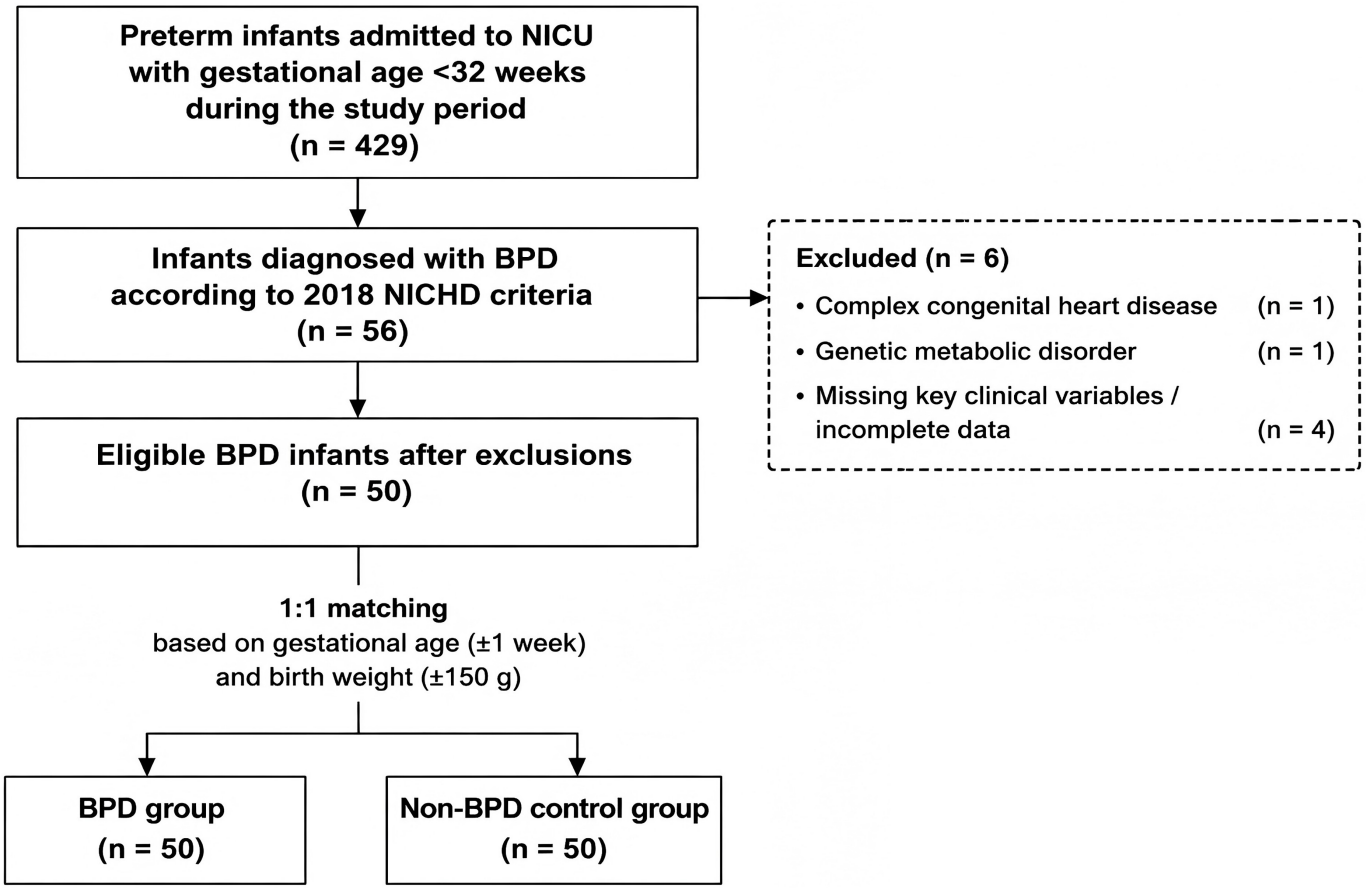
Study flow diagram.

The enrollment process of the study participants is illustrated in Figure 1. During the study period, 429 preterm infants with gestational age < 32 weeks were admitted to our NICU. Among them, 56 infants were diagnosed with BPD. Six infants were excluded according to predefined exclusion criteria, including one infant with complex congenital heart disease, one with a confirmed genetic metabolic disorder, and four infants with missing key clinical variables required for analysis or incomplete data that could potentially introduce bias. Finally, 50 infants with BPD were included in the study. Subsequently, 50 non-BPD controls were selected from concurrently admitted eligible infants using 1:1 matching based on gestational age and birth weight.

Baseline and maternal perinatal characteristics of both groups are presented in Table 1. No significant differences were observed between the BPD and non-BPD groups in neonatal characteristics, including sex, birth weight, gestational age, multiple gestation, in vitro fertilization status, small-for-gestational-age status, Apgar scores at 1 and 5 min, and antenatal corticosteroid exposure (all *P* > 0.05). Maternal characteristics, including maternal age and pregnancy-related conditions such as hypertensive disorders of pregnancy, gestational diabetes mellitus, intrauterine infection, hypothyroidism, cervical incompetence, intrahepatic cholestasis of pregnancy, gestational anemia, gestational thrombocytopenia, placental abruption, placenta previa, fetal distress, and meconium-stained amniotic fluid, were also comparable between groups (all *P* > 0.05).

**TABLE 1:** Baseline clinical characteristics of the two groups.

Variable	BPD group (*n* = 50)	Non-BPD group (*n* = 50)	*P*
Neonatal baseline characteristics			
Male sex, n (%)	31 (62.0)	34 (68.0)	0.529
Birth weight (g)	1186.66 ± 218.87	1184.80 ± 235.63	0.968
Gestational age (weeks)	29.60 ± 1.89	29.54 ± 1.70	0.866
Multiple gestation, n (%)	12 (24.0)	9 (18.0)	0.461
In vitro fertilization, n (%)	9 (18.0)	5 (10.0)	0.249
SGA, n (%)	5 (10.0)	7 (14.0)	0.538
Apgar score at 1 min	8.50 (7.50, 9.00)	9.00 (8.00, 9.00)	0.595
Apgar score at 5 min	9.00 (8.00, 10.00)	9.50 (8.00, 10.00)	0.705
Maternal characteristics			
Maternal age (years)	30.00 (29.00, 34.75)	29.50 (28.75, 33.25)	0.875
Hypertensive disorder of pregnancy, n (%)	6 (12.0)	6 (12.0)	1.000
Gestational diabetes mellitus, n (%)	15 (30.0)	9 (18.0)	0.242
Intrauterine infection, n (%)	7 (14.0)	3 (6.0)	0.317
Hypothyroidism, n (%)	9 (18.0)	5 (10.0)	0.387
Cervical incompetence, n (%)	18 (36.0)	11 (22.0)	0.186
Intrahepatic cholestasis of pregnancy, n (%)	3 (6.0)	0 (0.0)	0.242
Gestational anemia, n (%)	3 (6.0)	5 (10.0)	0.715
Gestational thrombocytopenia, n (%)	0 (0.0)	2 (4.0)	0.495
Placental abruption, n (%)	7 (14.0)	1 (2.0)	0.059
Placenta previa, n (%)	4 (8.0)	0 (0.0)	0.117
Fetal distress, n (%)	0 (0.0)	4 (8.0)	0.117
Meconium-stained amniotic fluid, n (%)	13 (26.0)	12 (24.0)	1.000
Antenatal corticosteroids, n (%)	39 (78.0)	35 (70.0)	0.362

BPD, bronchopulmonary dysplasia; SGA, small for gestational age.

### 3.2 Vital signs, perfusion index, and microcirculatory parameters

Between-group comparisons of vital signs and perfusion-related indices are shown in Table 2. Baseline heart rate, SpO2, and respiratory rate did not differ significantly between groups (all *P* > 0.05). Compared with the non-BPD group, infants in the BPD group had greater SpO2 variability and a lower PaO_2_/FiO_2_ ratio (both *P* < 0.05). Regarding peripheral perfusion, the BPD group had a significantly lower PI 7-day mean and a more negative PI trend slope, while the 7-day mean PVI and peak PVI were both higher (all *P* < 0.05). Capillary refill time, individual daily PI and PVI values, PI minimum, PI coefficient of variation, and PVI standard deviation did not differ significantly between groups (all *P* > 0.05).

**TABLE 2:** Comparison of vital signs, perfusion index, and microcirculatory parameters.

Variable	BPD group (*n* = 50)	Non-BPD group (*n* = 50)	*P*
Vital signs			
Baseline heart rate (beats/min)	125.94 ± 9.32	123.10 ± 9.83	0.141
Baseline SpO_2_ (%)	97.00 (95.00, 98.00)	97.00 (95.50, 98.50)	0.694
Respiratory rate (breaths/min)	51.00 ± 4.59	50.70 ± 4.37	0.739
SpO_2_ variability (%)	6.20 (4.88, 7.12)	4.90 (4.00, 6.60)	0.013
Capillary refill time (s)	2.88 ± 0.89	2.80 ± 1.00	0.704
PaO_2_/FiO_2_ ratio	412.00 (364.75, 463.75)	461.00 (422.50, 499.25)	0.004
Perfusion index (PI)			
PI day 1	1.35 ± 0.46	1.23 ± 0.48	0.102
PI day 2	1.17 (0.93, 1.40)	1.36 (0.93, 1.63)	0.118
PI day 3	1.17 ± 0.47	1.27 ± 0.42	0.242
PI day 4	1.16 ± 0.44	1.28 ± 0.35	0.138
PI day 5	1.20 (0.99, 1.41)	1.29 (1.01, 1.60)	0.228
PI day 6	1.19 ± 0.46	1.31 ± 0.46	0.168
PI day 7	1.21 ± 0.38	1.35 ± 0.45	0.082
PI 7-day mean	1.21 ± 0.17	1.29 ± 0.16	0.012
PI minimum value	0.64 (0.39, 0.86)	0.73 (0.57, 0.95)	0.185
PI coefficient of variation (%)	32.61 (23.30, 41.87)	28.52 (20.45, 34.51)	0.201
PI trend slope	−0.01 ± 0.07	0.03 ± 0.09	0.029
Pleth variability index (PVI)			
PVI day 1	27.91 ± 14.21	23.66 ± 11.93	0.108
PVI day 2	25.03 ± 10.82	21.83 ± 12.50	0.174
PVI day 3	20.68 (15.94, 30.39)	22.92 (14.88, 31.39)	0.717
PVI day 4	24.00 ± 10.01	22.46 ± 12.84	0.506
PVI day 5	28.16 ± 11.85	25.69 ± 11.53	0.295
PVI day 6	20.35 (10.20, 30.82)	18.60 (10.36, 26.42)	0.372
PVI day 7	22.50 (15.75, 29.27)	15.91 (12.20, 27.92)	0.051
PVI 7-day mean	25.45 (21.97, 28.29)	22.51 (19.15, 25.44)	0.005
PVI peak value	41.39 ± 8.43	37.86 ± 6.99	0.025
PVI standard deviation	10.51 ± 2.84	10.59 ± 3.03	0.986

BPD, bronchopulmonary dysplasia; PI, perfusion index; PVI, pleth variability index; SpO_2_, peripheral oxygen saturation.

### 3.3 Blood gas parameters and echocardiographic features

Blood gas findings are summarized in Table 3. Immediately postnatal blood gas values, including lactate, pH, BE-B, actual bicarbonate, PCO2, and PO2, were comparable between groups (all *P* > 0.05). Among the worst blood gas values obtained within the first 7 postnatal days, the BPD group had a significantly lower pH and actual bicarbonate level and a more negative BE-B relative to the non-BPD group, while peak lactate was markedly elevated (all *P* < 0.05). PCO2 and PO2 did not differ significantly (both *P* > 0.05). These findings indicate more pronounced abnormalities in acid–base balance and higher peak lactate levels among infants who subsequently developed BPD. Echocardiographic findings are detailed in Table 4. In terms of systolic function, the BPD group had significantly lower LVEF and LVFS. Regarding ventricular geometry, LVEDD, IVSd, and LVPWd were all significantly smaller in the BPD group (all *P* < 0.05). LVESD, Vmax, and pressure gradient were comparable between groups (all *P* > 0.05).

**TABLE 3:** Comparison of blood gas parameters between the two groups.

Variable	BPD group (*n* = 50)	Non-BPD group (*n* = 50)	*P*
Immediately postnatal blood gas			
Lactate (mmol/L)	1.80 (1.50, 2.60)	1.70 (1.50, 2.18)	0.282
pH	7.34 (7.21, 7.42)	7.32 (7.23, 7.41)	0.976
Base excess (BE-B, mmol/L)	−4.10 (−5.20, −1.48)	−3.80 (−4.97, −1.90)	0.476
Actual bicarbonate (mmol/L)	21.50 (17.60, 24.60)	22.60 (20.30, 25.90)	0.222
PCO2 (mmHg)	54.00 (48.75, 82.75)	50.50 (42.00, 58.00)	0.051
PO2 (mmHg)	93.00 (75.75, 113.00)	95.00 (78.00, 116.00)	0.221
Worst blood gas within postnatal 7 days			
pH	7.12 (7.11, 7.16)	7.19 (7.12, 7.22)	0.009
Base excess (BE-B, mmol/L)	−5.85 (−6.60, −3.93)	−5.25 (−6.17, −3.80)	0.025
Actual bicarbonate (mmol/L)	23.80 (22.10, 25.40)	25.90 (24.70, 26.50)	0.021
PCO_2_ (mmHg)	52.50 (50.00, 57.00)	51.00 (50.00, 54.00)	0.157
PO_2_ (mmHg)	76.00 (66.25, 88.00)	83.00 (69.00, 90.00)	0.220
Peak lactate (mmol/L)	4.20 (3.10, 5.15)	2.75 (1.70, 3.70)	< 0.001

BPD, bronchopulmonary dysplasia; BE-B, base excess in blood; PCO_2_, partial pressure of carbon dioxide; PO_2_, partial pressure of oxygen.

**TABLE 4:** Comparison of echocardiographic features between the two groups.

Variable	BPD group (*n* = 50)	Non-BPD group (*n* = 50)	*P*
LVEF (%)	67.15 ± 4.85	71.81 ± 4.17	< 0.001
LVFS (%)	34.64 ± 3.20	37.51 ± 4.35	< 0.001
LVEDD (mm)	13.18 ± 1.12	13.69 ± 1.19	0.032
LVESD (mm)	8.65 ± 0.98	8.60 ± 0.84	0.810
IVSd (mm)	2.62 ± 0.38	2.80 ± 0.41	0.030
LVPWd (mm)	2.01 ± 0.42	2.20 ± 0.47	0.035
Vmax (m/s)	2.28 (1.89, 2.54)	2.06 (1.68, 2.54)	0.228
Pressure gradient (mmHg)	18.19 (14.16, 23.70)	15.30 (6.83, 23.34)	0.129

BPD, bronchopulmonary dysplasia; LVEF, left ventricular ejection fraction; LVFS, left ventricular fractional shortening; LVEDD, left ventricular end-diastolic diameter; LVESD, left ventricular end-systolic diameter; IVSd, interventricular septal thickness in diastole; LVPWd, left ventricular posterior wall thickness in diastole.

### 3.4 Respiratory support characteristics and major comorbidities

Respiratory support characteristics and major comorbidities are detailed in Table 5. Compared with the non-BPD group, infants with BPD had a significantly higher requirement for invasive mechanical ventilation, a longer duration of invasive ventilation, and a higher peak FiO_2_ during the first postnatal week (all *P* < 0.05). No significant differences were observed in surfactant administration, CPAP use, patent ductus arteriosus, atrial septal defect, ventricular septal defect, or late-onset sepsis between the two groups (all *P* > 0.05).

**TABLE 5:** Respiratory support characteristics and major comorbidities.

Variable	BPD group (*n* = 50)	Non-BPD group (*n* = 50)	*P*
Surfactant administration, n (%)	44 (88.0)	46 (92.0)	0.741
IMV, n (%)	38 (76.0)	9 (18.0)	< 0.001
CPAP use, n (%)	50 (100.0)	48 (96.0)	0.495
Duration of IMV (days)	7.0 (0.0, 12.8)	1.0 (0.0, 3.0)	< 0.001
Peak FiO_2_ (%)	50.5 (44.3, 58.0)	36.5 (26.3, 44.8)	< 0.001
Patent ductus arteriosus, n (%)	43 (86.0)	40 (80.0)	0.595
Atrial septal defect, n (%)	38 (76.0)	34 (68.0)	0.504
Ventricular septal defect, n (%)	1 (2.0)	1 (2.0)	1.000
Late-onset sepsis, n (%)	4 (8.0)	3 (6.0)	1.000

BPD, bronchopulmonary dysplasia; CPAP, continuous positive airway pressure; IMV, invasive mechanical ventilation; FiO_2_, fraction of inspired oxygen; PDA, patent ductus arteriosus.

### 3.5 Univariate and multivariate logistic regression analyses

Candidate variables showing clinical differences between the BPD and non-BPD groups were entered into LASSO regression, which identified 13 variables with non-zero coefficients (Supplementary Table S1). To reduce multicollinearity while maintaining physiological interpretability, variables were grouped according to physiological domains, and the representative variable with the largest absolute LASSO coefficient was retained from each domain (all variance inflation factors < 1.1), covering peripheral perfusion, microcirculation, oxygenation, cardiac systolic function, cardiac structure, and acid–base/metabolic status. Because IMV and peak FiO_2_ are well-established indicators of respiratory disease severity and clinically relevant factors associated with BPD development, these two variables were prespecified and additionally included in the multivariable model irrespective of LASSO selection.

A Firth bias-corrected multivariable logistic regression model identified IMV aOR: 21.312, 95% CI: 2.108–36.296, *P* = 0.023), peak FiO_2_ (aOR: 1.404, 95% CI: 1.022–1.931, *P* = 0.036), lower PI trend slope (aOR: 0.041, 95% CI: 0.002–0.974, *P* = 0.048), lower PaO_2_/FiO_2_ ratio (aOR: 0.026, 95% CI: 0.001-0.848, *P* = 0.040), lower LVEF (aOR: 0.008, 95% CI: 0.000–0.602, *P* = 0.028), and thinner IVSd (aOR: 0.043, 95% CI: 0.002–0.933, *P* = 0.045) as independent predictors of BPD. The 7-day mean PVI and peak lactate did not remain independently associated with BPD after multivariable adjustment (Table 6).

**TABLE 6:** Univariate and multivariate logistic regression analysis of risk factors for BPD.

Variable	Univariate OR (95% CI)	*P*	Multivariate aOR (95% CI)	*P*
IMV	14.436 (5.466–28.071)	< 0.001	21.312 (2.108 –36.296)	0.023
Peak FiO_2_	1.170(1.098–1.245)	< 0.001	1.404 (1.022–1.931)	0.036
PI trend slope	0.630 (0.413–0.962)	0.032	0.041 (0.002–0.974)	0.048
PVI 7-day mean	1.778 (1.156–2.732)	0.009	2.899 (0.706 –11.906)	0.140
PaO_2_/FiO_2_ ratio	0.519 (0.332–0.812)	0.004	0.026 (0.001–0.848)	0.040
LVEF	0.297 (0.162–0.546)	< 0.001	0.008 (0.000–0.602)	0.028
IVSd	0.633 (0.415–0.965)	0.034	0.043 (0.002–0.933)	0.045
Peak lactate	2.637 (1.553–4.479)	< 0.001	21.395 (0.790–59.240)	0.069

BPD, bronchopulmonary dysplasia; OR, odds ratio; aOR, adjusted odds ratio; CI, confidence interval; IMV, invasive mechanical ventilation; PI, perfusion index; PVI, pleth variability index; LVEF, left ventricular ejection fraction; IVSd, interventricular septal thickness in diastole.

**FIGURE 2:**
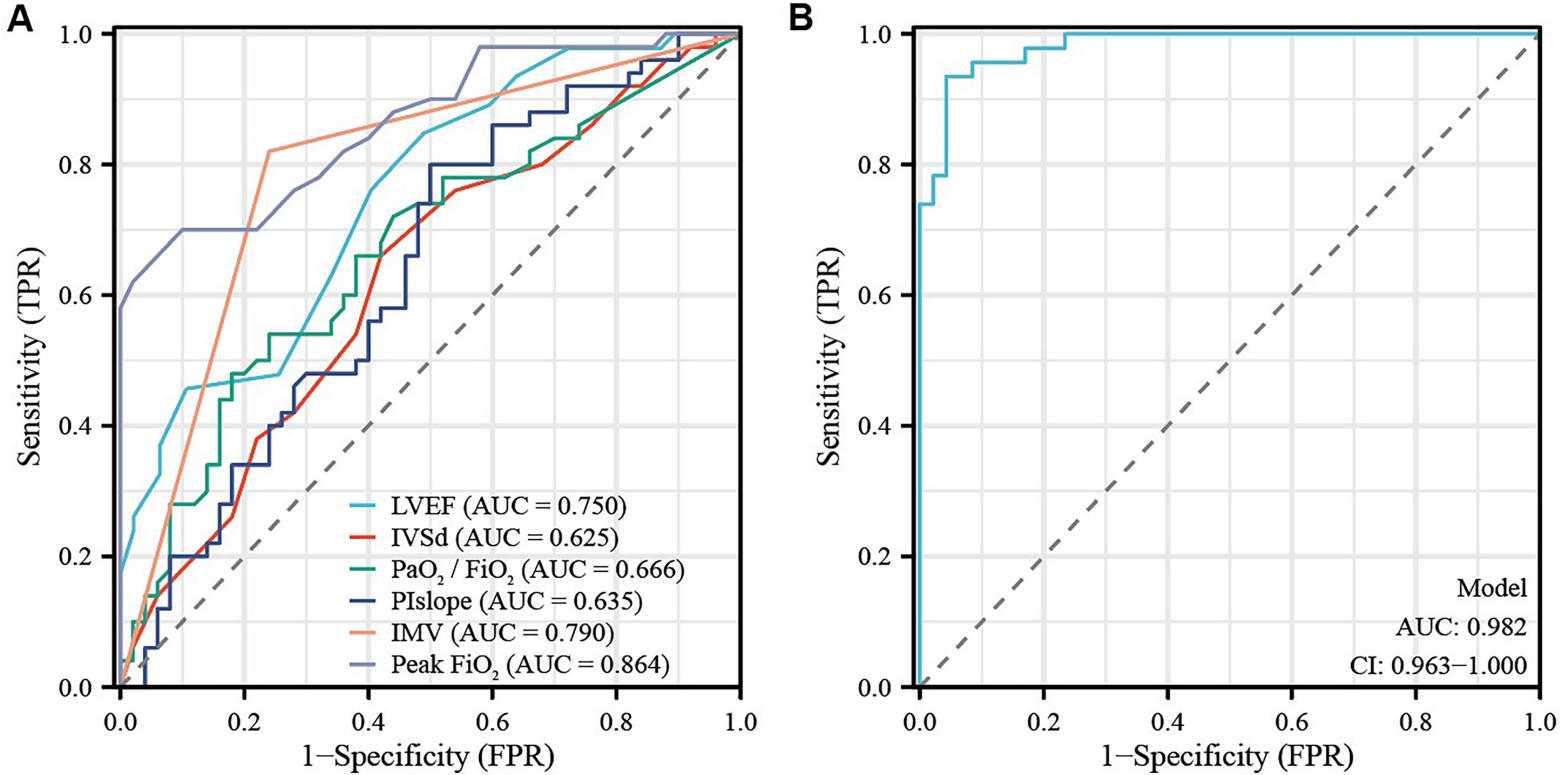
Receiver operating characteristic (ROC) curves for the prediction of BPD using **(A)** individual clinical variables and **(B)** the combined model. BPD, bronchopulmonary dysplasia.

The final six-variable model achieved an AUC of 0.982 (95% CI, 0.963–1.000), exceeding the discriminative performance of each individual predictor (individual AUC range: 0.625–0.864). Model calibration was satisfactory (Hosmer-Lemeshow χ^2^ = 2.05, *P* = 0.979), with a Nagelkerke *R*^2^ of 0.863. Internal validation using 1,000 bootstrap resamples demonstrated minimal optimism (0.012), corresponding to an optimism-corrected AUC of 0.970, indicating good model stability and limited overfitting. At the optimal Youden index threshold, the model achieved a sensitivity of 93.47% and a specificity of 94.62% (Figure 2 and Table 7).

**TABLE 7:** Predictive performance of individual and combined variables for BPD in preterm infants.

Variable	AUC	95% CI	Sensitivity (%)	Specificity (%)
PI trend slope	0.635	0.526–0.745	80.00	50.00
PaO_2_/FiO_2_	0.666	0.559–0.773	54.00	76.00
LVEF	0.750	0.653–0.847	84.78	51.06
IVSd	0.625	0.516–0.735	66.00	58.00
IMV	0.790	0.710–0.870	82.00	76.00
Peak FiO_2_	0.864	0.794–0.934	74.00	94.00
Combined model	0.982	0.963–1.000	93.47	94.62

BPD, bronchopulmonary dysplasia; AUC, area under the receiver operating characteristic curve; CI, confidence interval.

To further evaluate early respiratory trajectories, six BPD infants (12.0%) initially required only low-level respiratory support during the first postnatal week but subsequently required escalation of respiratory support after day 7. Among these infants, the model correctly identified five as high risk, corresponding to a sensitivity of 83.3% (5/6) in this subgroup. To further evaluate the influence of these delayed deterioration cases, a sensitivity analysis excluding the six infants was performed. The model maintained comparable discriminative performance, with an AUC of 0.980 (95% CI: 0.959-1.000), a sensitivity of 92.50%, and a specificity of 95.75%, further supporting the robustness of the first-week physiological profile.

## 4 Discussion

In this matched case-control study of preterm infants born at less than 32 weeks’ gestation, IMV, peak FiO_2_, a more markedly declining PI trend slope, a lower PaO_2_/FiO_2_ ratio, lower LVEF, and thinner IVSd were independently associated with subsequent BPD development. Because matching effectively balanced gestational age and birth weight, these associations are unlikely to be explained solely by differences in maturity. The combined model integrating established respiratory severity indicators with multi-system physiological biomarkers achieved excellent discrimination (AUC = 0.982), substantially outperforming any individual predictor. The independent association of IMV is consistent with its established role as a major determinant of BPD, whereas the persistence of the physiological biomarkers after adjustment indicates that they provide complementary information beyond respiratory support severity.

The independent association between lower LVEF and BPD is consistent with evidence that the immature myocardium is already compromised in the early stages of evolving lung disease. Prior studies have reported that right and left ventricular dysfunction within the first three postnatal weeks is associated with death and severe BPD in extremely preterm infants (7), and impaired left ventricular systolic and diastolic function has been documented in infants with established BPD (9). A plausible mechanism involves early pulmonary vascular disease increasing right ventricular afterload and subsequently impairing left ventricular filling and ejection through ventricular interdependence (6, 11). However, because dedicated echocardiographic markers of pulmonary hypertension and right ventricular function were not assessed, this mechanism remains speculative and requires confirmation using advanced functional echocardiography. The independent association between thinner IVSd and BPD is a novel finding. Unlike the septal hypertrophy typically observed in pulmonary hypertension associated with advanced BPD (12), infants who subsequently developed BPD had a thinner interventricular septum during early postnatal life. This may reflect impaired myocardial development or altered ventricular geometry, although the underlying mechanism remains speculative and warrants further investigation using advanced functional echocardiography.

The finding that a lower PaO_2_/FiO_2_ ratio independently predicted BPD is consistent with its role as a readily available bedside measure of gas exchange efficiency. Previous studies have demonstrated impaired gas exchange in BPD, mainly reflecting ventilation–perfusion mismatch and pulmonary parenchymal and vascular injury (10). Consistent with this, peak FiO_2_ also remained an independent predictor, indicating that impaired oxygenation and increased oxygen requirement provide complementary information for early BPD risk stratification. Comparable to the oxygenation findings, our perfusion results highlight the value of dynamic trends over single time-point measurements. Healthy preterm infants typically show a gradual rise in PI during the first postnatal week as peripheral perfusion matures (13–15); however, this increase was absent in infants who subsequently developed BPD, resulting in a flattened or negative PI trend slope. Previous studies have shown that lower PI is associated with reduced systemic perfusion and greater disease severity (16–18). Although the 7-day mean PVI was associated with BPD in the univariate analysis, it lost significance after multivariable adjustment, suggesting that the PI trend slope better reflects early microcirculatory maladaptation. Collectively, these findings support the clinical value of continuous perfusion trend monitoring over isolated measurements.

Peak lactate within the first postnatal week was significantly higher in infants who subsequently developed BPD, whereas immediately postnatal lactate did not differ between cases and controls. Lactate is a well-established biomarker of tissue hypoperfusion and anaerobic metabolism, and its elevation in neonates has been associated with increased morbidity and mortality across a range of clinical contexts (19–21). Although peak lactate did not remain an independent predictor after multivariable adjustment, its temporal increase during the first postnatal week likely reflects the cumulative burden of hypoperfusion and metabolic stress accompanying early BPD development. This finding suggests that lactate may serve as a marker of overall physiological instability rather than an independent determinant of BPD once respiratory and cardiovascular dysfunction are taken into account. This interpretation is consistent with emerging multi-omics evidence that neonatal diseases involve coordinated metabolic-immune interactions rather than isolated organ dysfunction (22).

Existing BPD prediction models have predominantly incorporated gestational age, birth weight, and evolving respiratory support variables, with relatively limited integration of physiological biomarkers (8, 9, 23). Concurrently, growing evidence has highlighted the contribution of early pulmonary vascular, cardiovascular, and microcirculatory dysfunction to BPD pathophysiology (6, 11). Against this background, the present study integrated representative bedside biomarkers across complementary physiological domains into a parsimonious model. In particular, the use of a 7-day PI trend slope captures dynamic microcirculatory adaptation rather than a static perfusion measurement (15). The final model integrated PI trend slope, PaO_2_/FiO_2_ ratio, LVEF, and IVSd with the established respiratory severity indicators IMV and peak FiO_2_. This six-variable physiology-based model achieved excellent discrimination and may provide complementary predictive information beyond respiratory support variables alone.

From a clinical perspective, all components of this BPD physiological profile are inexpensive, reproducible, and routinely available in neonatal intensive care, supporting its potential clinical applicability. Nevertheless, several limitations should be acknowledged. First, this was a single-center retrospective study with a relatively limited sample size. Although matching, penalized regression, and internal bootstrap validation were applied to minimize overfitting, the limited events-per-variable ratio constrained the inclusion of additional clinically relevant covariates, such as hemodynamically significant patent ductus arteriosus, duration of invasive mechanical ventilation, surfactant use, and late-onset sepsis, to preserve model stability. While sensitivity analyses yielded consistent findings, residual confounding cannot be completely excluded. Second, matching for gestational age and birth weight reduced baseline imbalance but precluded assessment of their independent predictive effects and may limit the generalizability of the model. In addition, the study population consisted predominantly of infants born between 28 and 31^+6^ weeks’ gestation; therefore, the model should be interpreted as an early risk stratification tool for this population rather than a universal screening strategy for all preterm infants born at < 32 weeks’ gestation. Third, echocardiographic and blood gas-derived variables were collected over different sampling windows, with blood gas variables represented by the worst values during the first postnatal week. Consequently, the integrated physiological profile reflects complementary information acquired at different stages of early postnatal adaptation rather than a single contemporaneous cardiopulmonary state. Finally, the model was intentionally developed using physiological data from the first postnatal week to facilitate ultra-early risk stratification. Although sensitivity analysis excluding infants with delayed respiratory deterioration after day 7 demonstrated consistent predictive performance, this early physiological snapshot may not fully capture late-onset phenotypes or the biological heterogeneity of BPD. Future prospective studies incorporating longer longitudinal monitoring and trajectory-based modeling are warranted to further refine prediction across different disease trajectories.

## 5 Conclusion

This study identifies an early bedside physiological profile integrating respiratory support severity (IMV and peak FiO_2_), PI trend slope, PaO_2_/FiO_2_ ratio, LVEF, and IVSd, which demonstrated excellent discriminative ability for early identification of infants at risk of BPD. By integrating respiratory support severity, oxygenation, peripheral perfusion, cardiac systolic function, and cardiac structure, this profile offers a physiology-based and clinically practical approach to early risk stratification in very preterm infants. Prospective, multi-center external validation in larger cohorts is now needed to confirm its predictive performance and to determine whether targeted interventions against these early biomarkers can meaningfully improve clinical outcomes.

## Data Availability

The original contributions presented in the study are included in the article/Supplementary material, further inquiries can be directed to the corresponding authors.
