# Supplementary material for: Early multi-system physiological biomarkers of bronchopulmonary dysplasia in preterm infants: a matched case-control study based on bedside monitoring
Source: Front Med (Lausanne). 2026 Jul 27;13:1902303. doi: 10.3389/fmed.2026.1902303 (PMC13454276; doi:10.3389/fmed.2026.1902303)
Supplement: Supplementary file 1 [file Table_1.docx]

**Supplementary Table S1. LASSO Coefficient Selection of Clinical Variables for BPD**

| **Rank** | **Variable** | **LASSO standardized coefficient** |
| --- | --- | --- |
| 1 | LVEF | −0.651 |
| 2 | LVFS | −0.548 |
| 3 | Peak lactate | +0.531 |
| 4 | Oxygenation index (PaO₂/FiO₂ ratio) | −0.377 |
| 5 | IVSd | −0.253 |
| 6 | LVPWd | −0.187 |
| 7 | PI trend slope | −0.173 |
| 8 | PVI 7-day mean | +0.137 |
| 9 | Worst pH (day 7) | −0.135 |
| 10 | PI 7-day mean | −0.110 |
| 11 | SpO_2_ variability | +0.026 |
| 12 | LVEDD | −0.012 |
| 13 | Worst BE-B (day 7) | −0.012 |
| 14 | Worst bicarbonate (day 7) | 0 |
| 15 | PVI peak value | 0 |

*LASSO, least absolute shrinkage and selection operator; BPD, bronchopulmonary dysplasia; LVEF, left ventricular ejection fraction; LVFS, left ventricular fractional shortening; IVSd, interventricular septal thickness in diastole; LVPWd, left ventricular posterior wall thickness in diastole; PVI, pleth variability index; PI, perfusion index; BE-B, base excess in blood; LVEDD, left ventricular end-diastolic diameter.*
